# Description of a new freshwater bacterium *Aquirufa regiilacus* sp. nov., classification of the genera *Aquirufa*, *Arundinibacter*, *Sandaracinomonas*, and *Tellurirhabdus* to the family *Spirosomataceae*, classification of the genus *Chryseotalea* to the family *Fulvivirgaceae* and *Litoribacter* to the family *Cyclobacteriaceae*, as well as classification of *Litoribacter alkaliphilus* as a later heterotypic synonym of *Litoribacter ruber*

**DOI:** 10.1007/s00203-023-03801-8

**Published:** 2024-01-27

**Authors:** Alexandra Pitt, Stefan Lienbacher, Johanna Schmidt, Meina Neumann-Schaal, Jacqueline Wolf, Martin W. Hahn

**Affiliations:** 1 Research Department for Limnology, Universität Innsbruck, Mondseestrasse 9, 5310 Mondsee, Austria; 2 Chemical Analytics and Metabolomics, Leibniz Institute DSMZ-German Collection of Microorganisms and Cell Cultures GmbH, Braunschweig, Germany

**Keywords:** *Aquirufa*, *Spirosomataceae*, *Cytophagaceae*, Freshwater, Genome, Genome size

## Abstract

Strains LEOWEIH-7C^T^ and LEPPI-3A were isolated from the Leopoldskroner Weiher, a lake located in the city of Salzburg, Austria. 16S rRNA gene similarities and phylogenetic reconstructions with 16S rRNA gene sequences as well as based on genome sequences revealed that the new strains belong to the *A. antheringensis* branch of the genus *Aquirufa*. Calculated whole-genome average nucleotide identity (gANI) and digital DNA–DNA hybridization (dDDH) values with the closely related type strains showed that the two strains represent a single new species. The strains grew aerobically and chemoorganotrophically, and the cells were rod shaped, on average 0.8 µm long and 0.3 µm wide, red pigmented and motile by gliding. The genome size of both strains was 2.6 Mbp and the G+C value was 41.9%. The genomes comprised genes predicted for the complete light-harvesting rhodopsin system and various carotenoids. We proposed to establish the name *Aquirufa regiilacus* sp. nov. for strain LEOWEIH-7C^T^ (=DSM 116390^T^ = JCM 36347^T^) as the type strain. Strain LEPPI-3A (=DSM 116391 = JCM 36348) also belongs to this new species. The calculated genome-based phylogenetic tree revealed that *Aquirufa* and some other genera currently allocated in the family *Cytophagaceae* need a reclassification. *Aquirufa*, *Arundinibacter*, *Sandaracinomonas*, and *Tellurirhabdus* should be designated to the family *Spirosomataceae*, the genus *Chryseotalea* to the family *Fulvivirgaceae*, and the genus *Litoribacter* to the family *Cyclobacteriaceae*. Furthermore, based on calculated gANI and dDDH values, *Litoribacter alkaliphilus* should be reclassified as a later heterotypic synonym of *Litoribacter ruber*.

**Supplementary Information:**

The online version contains supplementary material available at 10.1007/s00203-023-03801-8.

## Introduction

The genus *Aquirufa* was first described in 2019 (Pitt et al. 2019) as belonging to the family *Cytophagaceae* of the phylum *Bacteroidota*. Due to genome-based phylogenetic analyses, it was known that the family *Cytophagaceae* with its members showed polyphyly at that time (Hahnke et al. 2016). In 2019 it was proposed to split the family *Cytophagaceae* into three distinct families (García-López et al. 2019). Besides *Cytophagaceae* and *Flexibacteraceae*, the latter validated in 2020 (Oren and Garrity 2020), the authors recommended using the validly published name *Spirosomaceae* proposed in 1978 (Larkin and Borrall 1978) and orthographically corrected in 2022 to *Spirosomataceae* (Arahal et al. 2022). Some genera were not considered in the reorganization of the family *Cytophagaceae*, because they were described at the same time or near after. Our phylogenetic reconstructions based on genome sequences showed that the genus *Aquirufa* and some other genera, all currently allocated in the family *Cytophagaceae*, need a reclassification. Furthermore, the genomic tree and subsequently calculated whole-genome average nucleotide identity (gANI) values revealed that *Litoribacter alkaliphilus* is a later heterotypic synonym of *Litoribacter ruber*.

The family *Spirosomataceae* comprises Gram-negative, rod-forming, aerobic or facultatively anaerobic, non-spore-forming and pigmented bacteria with variable motility; the major menaquinone is menaquinone-7 and the major polar lipid is phosphatidylethanolamine (García-López et al. 2019).

The genus *Aquirufa* is characterized by aerobic, chemoorganotrophic, rod-shaped, red-pigmented freshwater bacteria with gliding motility (Pitt et al. 2019) including at the time of writing seven species with validly published names (Pitt et al. 2019, 2020, 2022; Sheu et al. 2020). These bacteria seem to occur all over the world in various standing and running freshwater habitats (Pitt et al. 2022). Closely related to the genus *Aquirufa* is the genus *Sandaracinomonas* with the only species *Sandaracinomonas limnophila*, which was also isolated from a freshwater habitat (Chen et al. 2020). Genome-based phylogenetic trees showed that the members of the genus *Aquirufa* form two lineages (Pitt et al. 2022). Species of the *A. antheringensis* branch (*A. antheringensis*, *A. lenticrescens*) are characterized by genome sizes of around 2.5 Mbp and G+C contents of around 42%. The *A. nivalisilvae* branch (*A. nivalisilvae*, *A. aurantiipilula*, *A. ecclesiirivi*, *A. beregesia*, *A. rosea*) consists of species with genome sizes around 3 Mbp and G+C contents around 38%.

Within a citizen science project, we searched for new *Aquirufa* strains in freshwater habitats in Salzburg and Upper Austria. The habitats were sampled and bacterial cultures belonging to the genus were obtained. Two strains, LEOWEIH-7C^T^ and LEPPI-3A, both isolated from water samples of a lake located in the city of Salzburg, represented a new species of the *A. antheringensis* branch.

So, we describe here the new species *Aquirufa regiilacus* sp. nov. with its type strain LEOWEIH-7C^T^. In addition, we propose the classification of *Aquirufa* and five other genera, which are currently placed in the family *Cytophagaceae*, to appropriate families and the reclassification of *Litoribacter alkaliphilus*.

## Materials and methods

### Home habitat and isolation

Strains LEOWEIH-7C^T^ and LEPPI-3A originated from an artificial lake called Leopoldskroner Weiher in the city of Salzburg. This lake with an area of 13 ha was created more than 500 years ago, probably as a retention reservoir. Later the castle Leopoldskron was built near the shore, which is today together with the pond and the surrounding park under a preservation order. Strains LEOWEIH-7C^T^ and LEPPI-3A were obtained from different water samples, both were taken from the surface water in November 2022 at the geographic coordinates 47.78326 N 13.04111 E and 47.78445 N 13.03846 E, respectively. Measurements revealed a neutral pH value for both samples and a conductivity of 399 µS cm^−1^ and 274 µS cm^−1^, respectively.

The isolation procedure started with filtering the water samples through 0.65 µm pore-size membrane filters and spreading the filtrate afterward on nutrient broth soytone yeast extract (NSY) agar plates (Hahn et al. 2004). We searched for colonies with red pigmentation, which is a characteristic of *Aquirufa* strains. They were picked and transferred into liquid NSY medium. Sanger sequencing of the marker gene *gyrB* (B subunit of the DNA gyrase) (Pitt et al. 2022) was used to search for strains representing new species. The candidate strains were obtained as pure cultures by alternating cultivation on NSY agar plates and in liquid NSY medium and subsequently stored in NSY medium with 15% glycerol at −80 °C. The strains were genome-sequenced and gANI values were calculated to verify the preliminary estimations.

### Phenotypic and chemotaxonomic characterization

For the phenotypic and chemotaxonomic investigations, the same methods were used as described previously (Pitt et al. 2022). To reveal the temperature range of growth occulated NSY agar plates were exposed to increasing temperatures. We started at 5 °C and increased the temperature until no growth was observed. Anaerobic growth was tested in an anaerobic chamber by the use of standard NSY agar plates, and NSY plates supplemented with 2 g l^−1^ NaNO_3_, for testing NaCl tolerance served agar plates with various NaCl concentrations (0.1% w/v steps). Cell dimensions were measured with an epifluorescence microscope (UV filter). For that purpose, liquid cultures were fixed with 2% paraformaldehyde and stained with 4′,6-diamidino-2-phenylindole (DAPI). Motility of the strains was tested on soft agar plates (1 g l^−1^ yeast extract, 0.1 g l^−1^ K_2_HPO_4_, and 2.0 g l^−1^ agar). 20 µl of a well-growing culture was placed in the middle of standard NSY plates and on soft agar plates, incubated at 21 °C, and observed for one week. For chemotaxonomic characterization, the composition of cellular fatty acids, polar lipids, and respiratory quinones were analyzed. For this purpose, cells were cultured in liquid NSY medium at room temperature and harvested after three days by centrifugation. To determine the cellular fatty acid composition, biomass was saponified, methylated, and subsequently analyzed on an Agilent Technologies 6890 N instrument coupled to a flame ionization detector following the protocol described by Sasser (Sasser 1990). Equivalent chain length values were calculated analogously to the Microbial Identification System Sherlock to provide the peak naming according to the TSBA6 database. The extract was analyzed in addition by gas chromatography/mass spectrometry (GC/MS) to identify the fatty acids (Vieira et al. 2021). Double bond positions were determined by further derivatization to dimethyl disulfide adducts and following GC/MS analysis (Moss and Lambert-Fair 1989). The polar lipids were extracted and analyzed following the description of Tindall (Tindall 1990a, b) and the methods of Bligh and Dyer (Bligh and Dyer 1959). Two-dimensional silica gel thin-layer chromatography served to separate the polar lipids. The total lipids were detected by the use of dodecamolybdophosphoric acid (Dmp) and specific functional groups by using α-naphthol, ninhydrin, and molybdenum blue. Respiratory quinones were extracted by solid-phase extraction for analyses served reversed-phase HPLC coupled to a diode array detector as well as a high-resolution mass spectrometer. This method was described previously (Vieira et al. 2021).

### Genomic characterization

For DNA extraction and genome sequencing, the method described by Hoetzinger et al. (Hoetzinger et al. 2017) was used. For this purpose, a shotgun library was paired-end sequenced with 2 × 150 bp (Illumina NovaSeq). The software SPAdes version 3.13.1 (Bankevich et al. 2012) was used for de novo genome assembly. The nucleotide coverage was calculated based on the k-mer coverage received from SPAdes by using the formula: nucleotide coverage = k-mer coverage * read length/(read length−k-mer length + 1). The software CheckM (Parks et al. 2015) provided by the online platform Galaxy Protologger (Hitch et al. 2021) was utilized for a quality check of the obtained genome sequences. Both genomes were annotated by the NCBI Prokaryotic Genome Annotation Pipeline (Tatusova et al. 2016) and the RAST annotation server (Aziz et al. 2008) and deposited at DDBJ/ENA/GenBank databases. They were also incorporated into the Integrated Microbial Genomes and Microbiomes Expert Review (IMG/MER) database (Chen et al. 2019) for further annotation. The IMG/MER tool Phylogenetic Profiler for Single Genes served for detection of the presence or absence of homologous protein-encoding genes in the regarded genomes. The settings were minimum 30% identity and max. E-value 1e^−5^. These data formed the basis of a Venn diagram, which was constructed with the online tool https://eulerr.co (Larsson 2021). The tool SEED viewer (Overbeek et al. 2005) was used for determining N50 and L50 values and an amino acid sequence-based comparison of the genomes of strains LEOWEIH-7C^T^ and LEPPI-3A with the type strains of *A. lenticrescens* and *A. antheringensis*. The gANI and corresponding alignment fraction (AF) values were calculated with the IMG/MER system (Chen et al. 2019). Both parameters were obtained for all possible pairs involving all *Aquirufa* type strains. Additionally, the Type (Strain) Genome Server (Meier-Kolthoff et al. 2021) served for the determination of digital DNA–DNA hybridization (dDDH) values. The online tool Average Amino Acid Identity calculator from the Environmental Microbial Genomics Laboratory (Rodríguez-R and Konstantinidis 2016) served for the calculation of whole-genome (proteome) average amino acid identity (gAAI) values.

### Phylogenetic reconstructions

**Fig. 1:**
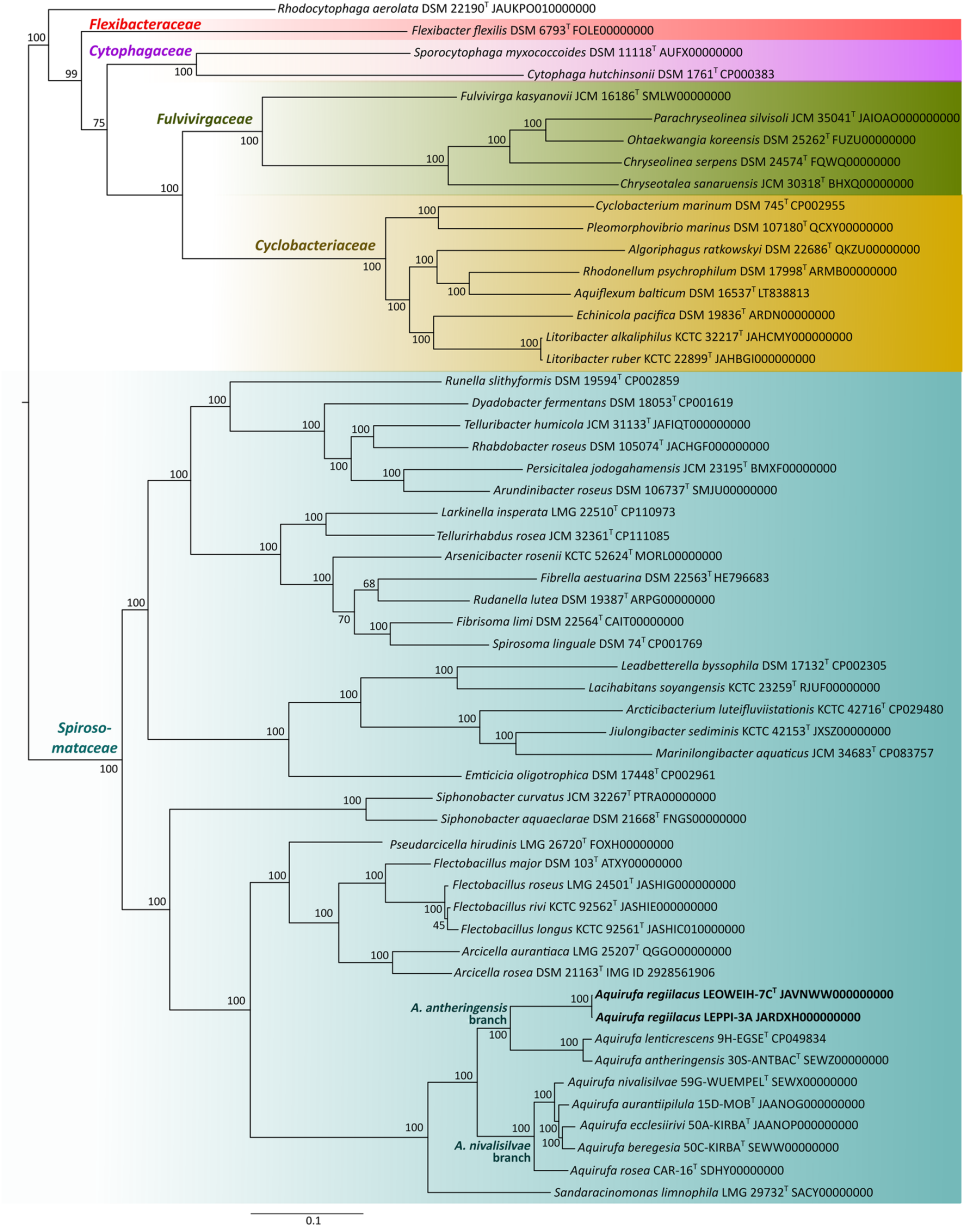
Midpoint rooted phylogenomic RAxML tree calculated with amino acid sequences obtained from 119 single-copy genes from all available genomes of the taxa from Figure S1. The classification of the genera into families (colored letters) is based on their position in the tree. Bar, 0.1 substitutions per nucleotide position (colour figure online)

Phylogenetic reconstructions were performed by using almost full-length sequences of the 16S rRNA gene as well as by utilizing amino acid sequences of 119 single-copy marker genes (Parks et al. 2018) based on genome sequences. For the phylogenetic tree based on 16S rRNA gene sequences (Figure S1), at least all type species of the genera listed by the LPSN database (Meier-Kolthoff et al. 2021) for the families *Cytophagaceae*, *Spirosomataceae*, *Flexibacteraceae* and *Fulvivirgaceae*, as well as a selection of the type species of the family *Cyclobacteriaceae*, were included. The software MEGA X (Kumar et al. 2018) was used to align the sequences, and a neighbor-joining tree was constructed with the parameters Kimura 2 model (Kimura 1980), invariant sites, gamma-distributed (5 categories), and 1000 bootstrap replicates. All strains from Figure S1 with available genome sequences were considered for the phylogenetic tree based on genome sequences (Fig. 1). A RAxMl tree was calculated with amino acid sequences of 119 protein-encoding genes (Parks et al. 2018). One of the 120 genes recommended by Parks et al., i.e., the protein family TIGR0009, was not found in all considered genomes and therefore omitted. MAFFT version 7 (Katoh et al. 2005) served to align the concatenate amino acid sequences. GBLOCKS version 0.91b (Castresana 2000) was used to filter out highly variable positions of the alignment. The result was a reduction of the alignment fraction from 50665 to 36179 amino acid positions (in 485 selected blocks). This represented 71% of the original alignment positions. The CIPRES Science Gateway version 3.3 (Miller et al. 2010) served to construct a RAxML tree (Stamatakis 2014) with 100 bootstrap replicates.

## Results and discussion

### Phenotypic and chemotaxonomic analyses

The phenotypic features of the type strain are shown in Table 1. The results for strain LEPPI-3A were very similar and are therefore not listed. The fatty acid composition of strain LEOWEIH-7C^T^ is presented in Table S1, the patterns of the polar lipids in Figure S2. When growing exclusively in a liquid medium for several weeks, cultures of strain LEOWEIH-7C^T^ became less colored. After spreading these paler cultures on agar plates, white colonies occurred among the normal red ones and could be purified. It seemed that this phenomenon was caused by a mutation.

**Table 1:** Discriminating features of the type strain of the new species and the type strains of the genus *Aquirufa* of the *A. antheringensis* branch

	1	2	3
Mean cell length (µm)	0.8	1.7	1.2
Mean cell width (µm)	0.3	0.6	0.5
Temperature range for growth (°C)	5–32 (w)	5–32 (w)	5–31 (w)
NaCl tolerance (%, w/v)	0–0.3	0–0.3 (w)	0–0.1 (w)
*Respiratory quinones*			
Menaquinone-6	6%	TR	−
Menaquinone-8	−	−	TR
*Fatty acids*			
C_15:1_ω4c^a^	4.4	2.4	−
Iso-C_17:1_ω7c^b^	1.2	−	2.1
anteiso-C_15:1_ω7c	1.7	−	−
Iso-C_15:1_ω5c	1.8	−	0.5
Iso-C_15:1_ω7c	1.8	−	−
Iso-C_16:0_3-OH	−	0.2	1.2
Iso-C_17:0_3-OH	−	1.1	1.1
*Polar lipids*			
Unidentified aminolipids	1	−	−
Unidentified aminophospholipids	1	2	3
Unidentified polar lipids	3	4	2

1, *A. regiilacus* sp. nov. LEOWEIH-7C^T^; 2, *A. antheringensis* 30S-ANTBAC^T^; 3, *A. lenticrescens* 9H-EGSE^T^. All strains had in common as follows: cell morphology: rods, liquid culture (NSY): red–orange suspension, pigmentation colonies: red, motility on soft agar: +, anaerobic growth: −, major respiratory quinone: menaquinone-7, identified polar lipid: phosphatidylethanolamine. Only the differentiating fatty acids are listed. The whole fatty acid composition of the new strain is shown in Table S1. −, negative; +, positive; w, weak; TR, traces

Data in columns 2 (Pitt et al. 2019) and column 3 (Pitt et al. 2022) were published partly previously, but elevated under the same conditions in the same lab. MIDI system: ^a^unknown 14.959; ^b^summed feature 9

Table 1 indicates the features which distinguished the new type strain from the closely related type strains of the *A. antheringensis* branch. The cell size of the new strain was smaller than the cell dimensions of the related strains, as well as the patterns of the fatty acids and the polar lipids differed in some details.

### Phylogenetic analyses

BLAST analyses (Johnson et al. 2008) of strains LEOWEIH-7C^T^ and LEPPI-3A revealed 16S rRNA gene sequence similarities with the type strains of *A. antheringensis* and *A. lenticrescens* of 99.42% and 99.47%, respectively. It is known that *Aquirufa* species within the *A. antheringensis* branch and the *A. nivalisilvae* branch, respectively, have identical or nearly identical 16S rRNA gene sequences (Pitt et al. 2022). The phylogenetic reconstruction based on 16S rRNA gene sequences (Figure S1) could neither resolve the relationships between the *Aquirufa* species nor was suitable for assignment of the incorporated genera to a family. Nevertheless, the tree revealed the affiliation of the two new strains with *A. antheringensis* and *A. lenticrescens*. The whole-genome-based phylogenetic reconstructions (Fig. 1) confirmed an affiliation and pointed out a relatively far phylogenetic distance between the new strains and the type strains of the two above-mentioned species.

In addition, the genome-based tree (Fig. 1) showed that the species of the genera *Aquirufa*, *Arundinibacter, Sandaracinomonas*, and *Tellurirhabdus* were placed on the branch of the family *Spirosomataceae*, the species of the genus *Litoribacter* on the branch of the family *Cyclobacteriaceae*, and the species of the genus *Chryseotalea* on the branch of the family *Fulvivirgaceae*. The branch of the genus *Rhodocytophaga* was strongly separated from the mentioned families. Recently, the new family ‘*Rhodocytophagaceae*’ including *Rhodocytophaga* and a newly described genus ‘*Xanthocytophaga*’ was proposed (Zhang et al. 2023) but so far not validated. The two species of the genus *Litoribacter*, *L. ruber* and *L. alkaliphilus*, possessed very short branches.

### Genomic analyses

**Fig. 2:**
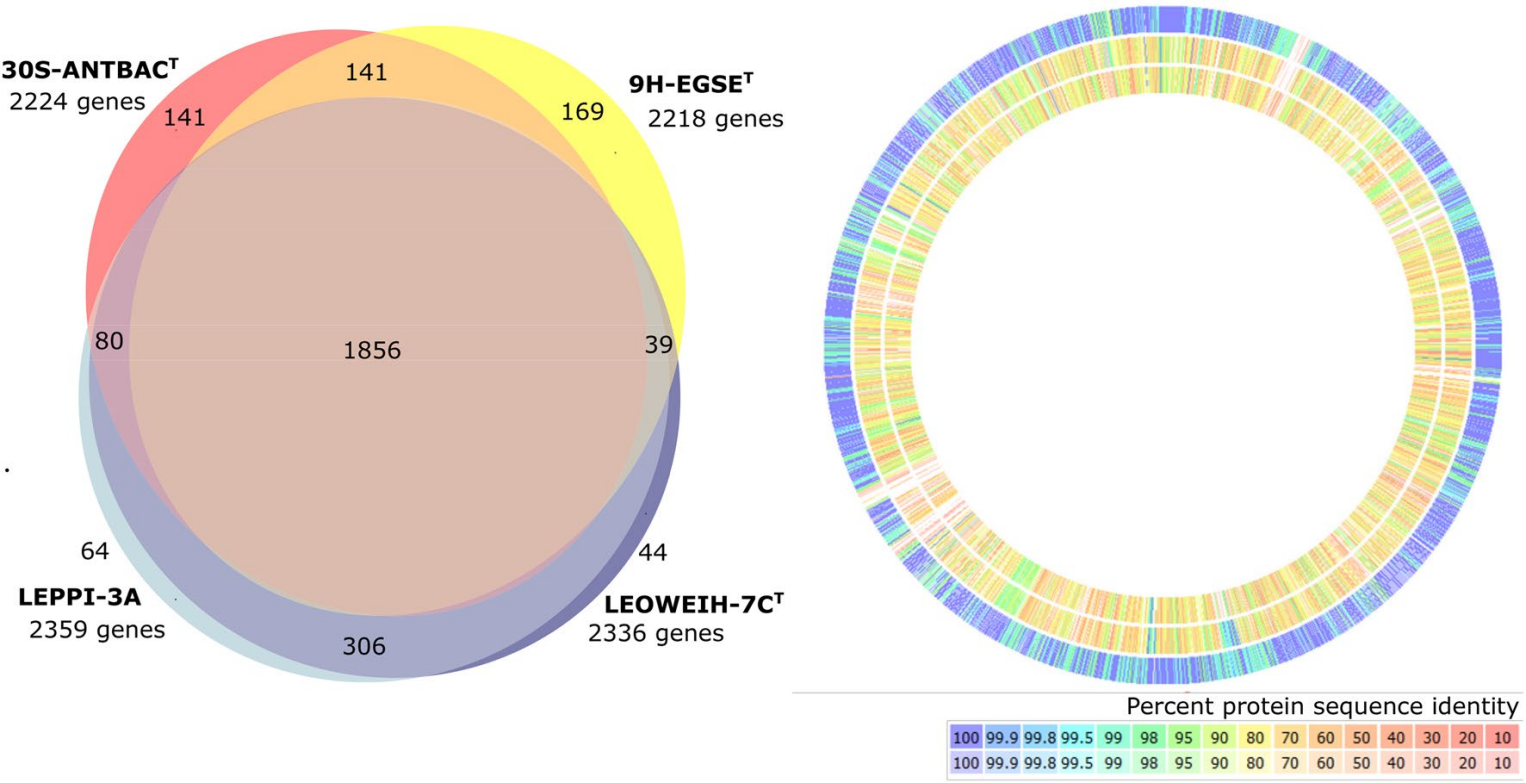
Genome sequence-based comparison of the two new strains (LEOWEIH-7C^T^ and LEPPI-3A) and the type strains of the closely related species (*A. antheringensis*, 30S-ANTBAC^T^ and *A. lenticrescens*, 9H-EGSE^T^). Left: Venn diagram of the protein-coding genes. Right: Protein sequence identities of strain LEOWEIH-7C^T^ compared with strain LEPPI-3A (outer circle), with strain 9H-EGSE^T^ (middle circle) and strain 30S-ANTBAC^T^ (inner circle), upper scale: bidirectional best hits, lower scale: unidirectional best hits

The results of genome sequencing were in the case of strain LEOWEIH-7C^T^ 12 contigs with a coverage value of 598x, N50 289 kbp, L50 2 and in the case of strain LEPPI-3A 40 contigs with a coverage of 470x, N50 291 kbp, L50 4, and the genome size of both genomes was 2.6 Mbp. The authenticity of the genomes was tested with Sanger sequences obtained from sequencing the 16S rRNA gene and the *gyrB* gene. Quality checks with CheckM revealed for both strains genome completeness of 96.3% and detected no genome contamination, and the genome sequence similarity of the two genomes was 99.1%. A comparison of the genome sequences of the novel strains and the type strains of nearly related species is given in Fig. 2. Regarding the homologous protein-coding genes (Fig. 2, left), approximately 80% of the genes were found in all three species. A little more than 400 genes were found exclusively in the new species (blue area). Even if considering only one of the two new strains to ensure equality, the new species had twice as many exclusive genes as the type strains of the nearest related species. This corresponded with the genome-based phylogenetic tree (Fig. 1), which revealed a wide distance between the branch of the new species and the nearest related *Aquirufa* species. Comparisons of the protein sequences (Fig. 2, right) consolidate this impression. The amino acid sequence similarities of the proteins in comparison of strain LEOWEIH-7C^T^ and the type strain of the closely related species were relatively low and corresponded with the average value (gAAI) of around 69%.

The pairwise calculated gANI values of the strains LEOWEIH-7C^T^ and LEPPI-3A and the type strains of the nearest related *Aquirufa* species (Table 2) were 77.3% and 78.0%, respectively, with relatively low AF values of around 50%. They ranged clearly under the accepted threshold of 95–96% utilized to delineate two prokaryotic species (Konstantinidis et al. 2006; Chun et al. 2018). The calculated dDDH values varied between 20.1 and 21.1% and were also far under the established threshold of 70% (Chun et al. 2018). On the other hand, the calculated values of 99.1% and 92.3%, respectively (Table 2), showed that strain LEOWEIH-7C^T^ and LEPPI-3A belong to the same species. The calculated gANI as well as dDDH values of the type strains of *L. alkaliphilus* and *L. ruber* of 98.4% and 85.8%, respectively, indicated clearly that they should belong to the same species. The differentiation of *L. alkaliphilus* from *L. ruber* as a new species was based on a lab DNA–DNA relatedness value of 23 ± 1% (Subhash et al. 2013), which is far below the accepted threshold. So, it is unclear what caused the wrong estimation.

**Table 2:** Genomic traits of the two new strains and the type strains of the genus *Aquirufa* of the *A. antheringensis* branch. The IMG/MER ID numbers of the mentioned genes are shown in Table S2

	1	2	3	4
IMG/MER ID number	8023692520	8014893578	2816332120	2857132225
Genome size (Mbp)	2.6	2.6	2.5	2.5
DNA G+C (%)	41.9	41.9	42.6	42.2
Coding density	94.1	94.0	94.8	95.0
Protein-coding genes	2336	2359	2266	2218
gANI with LEOWEIH-7C^T^ (%)	100	99.1	78.0	77.3
Corresponding average AF (%)	100	96.4	50.0	47.9
gANI with LEPPI-3A (%)	99.1	100	78.0	77.3
Corresponding average AF (%)	96.4	100	50.2	48.0
gAAI with LEOWEIH-7C^T^	100	97.7	69.0	67.7
gAAI with LEPPI-3A	97.4	100	68.6	67.4
dDDH with LEOWEIH-7C^T^ (d_4_, %)	100	92.3	21.1	20.2
dDDH with LEPPI-3A (d_4_, %)	92.3	100	20.8	20.1
*Genes predicted for*				
Bacteriorhodopsin (COG5524)	+	+	+	+
β-Carotene 15,15′-monooxygenase (TIGR03753)	+	+	+	+
Synthesis of β-carotene (KEEG map00906)	+	+	+	+
Nitrate reductase, assimilatory (EC:1.7.7.2)	+	+	+	−
Nitrite reductase, assimilatory (EC:1.7.1.15)	+	+	+	−
MFS transporter: nitrate/nitrite (COG2223)	+	+	+	−
Catalase-peroxidase (EC:1.11.1.21)	+	+	+	+
Cytochrome *c* peroxidase (EC:1.11.1.5)	2	2	+	+
Cytochrome *c* oxidase	+	+	+	+
Cytochrome *c* oxidase cbb3 type	+	+	+	+
Endoglucanase, cellulase (EC:3.2.1.4)	+	+	−	+
Rubredoxin (COG1773)	+	+	+	−
Genes involved in gliding motility	10	10	14	16
Genes for PorP/SprF proteins, type IX secretion system (TIGR03519)	6	6	5	6

1, LEOWEIH-7C^T^; 2, LEPPI-3A; 3, *A. antheringensis* 30S-ANTBAC^T^; 4, *A lenticrescens* 9H-EGSE^T^

Like all so far described *Aquirufa* strains, the new strains contained genes predicted for the whole glycolysis, citrate cycle, and pentose phosphate pathway as well as the biosynthesis of the carotenoids zeta-carotene, lycopene, beta-carotene, and astaxanthin. Some genes which were detected in all or some of the strains of the *A. antheringensis* branch are listed in Table 2. As all strains belonging to this branch, the new strains owned genes putatively encoding for the entire light-harvesting bacteriorhodopsin/retinal system. Ten genes were predicted in the IMG/MER system for gliding motility and six genes for the Type IX secretion system membrane protein PorP/SprF, which is confined to the phylum *Bacteroidota* and could also be involved in gliding motility (McBride 2019).

### Ecology of the new species

Two additional cultures, which were screened by sequencing the *gyrB* gene, were likely belonging to the proposed new species. They originated from the lake Hallstättersee and the river Vöckla, both located in Upper Austria (data not shown). Therefore, the new species seems to dwell in both standing and running freshwater systems. Since the 16S rRNA gene sequence similarities of the new species and the two closely related species (see above) are very high, BLAST searches with the 16S rRNA gene sequence are unsuitable for detecting the new species.

It is unclear if the species appears in the open water, free or attached to particles, or in the sediment. While the water samples from lake Leopoldskroner Weiher were taken from the lakeside, the sample from lake Hallstättersee originated from the water column, so a pelagic lifestyle is most probably. The capacity for gliding motility suggests at least a temporary association with particles. The genome size of 2.6 Mbp is quite small but in the upper range of genome sizes of typical pelagic freshwater bacteria (Chiriac et al. 2023), which is regarded as an adaption to a pelagic lifestyle. The same applies to the very high coding densities and the comparatively low G+C contents (Table 2) of the genomes of the new strains and the very small cell size. The occurrence of the light-harvesting rhodopsin system is generally discussed as an adaption of freshwater bacteria to low nutrient conditions (Chiriac et al. 2023), in any case, it is a hint on the occurrence of the new species in upper water layers, where enough light is available.

## Conclusion

The calculated 16S rRNA gene identity values showed that strains LEOWEIH-7C^T^ and LEPPI-3A belong to the genus *Aquirufa*. The calculated gANI, gAAI, and dDDH values indicated clearly that the strains represent a new species of the genus *Aquirufa*. The phylogenetic reconstructions based on genome sequences (Fig. 1) and the phenotypic and chemotaxonomic features of the type strain support this finding. For the new species with the type strain LEOWEIH-7C^T^, we propose the name *Aquirufa regiilacus* sp. nov.

In addition, based on the genomic phylogenetic tree of Fig. 1, we propose the reclassification of the genera *Aquirufa*, *Arundinibacter*, *Sandaracinomonas*, and *Tellurirhabdus* to the family *Spirosomataceae*, and the genus *Chryseotalea* to the family *Fulvivirgaceae*, as well as the genus *Litoribacter* to the family *Cyclobacteriaceae*. According to the calculated gANI and dDDH values, we propose to classify *Litoribacter alkaliphilus* as a later heterotypic synonym of *Litoribacter ruber*.

## Description of *Aquirufa regiilacus *sp. nov. (re.gi.i.la’cus. L. masc. n. *regius*, royal; L. masc. n. *lacus*, lake; N.L. gen. n. *regiilacus*, of a royal lake)

Cells form rods, about 0.3 µm wide and 0.8 µm long. Colonies grown on NSY or R2A agar are circular, and convex with a smooth surface; they are bright red, in older stages dark red pigmented. Liquid cultures (NSY or R2A medium) have a red–orange coloring. Cells show motility on soft agar. Growth occurs in 0–0.3% (w/v) NaCl and at 5–32 °C. Major fatty acids are iso-C_15:0_, C_16:1_ω7c and anteiso-C_15:0_. Polar lipids are three unidentified polar lipids, one unidentified aminophospholipid, one unidentified aminolipid, and phosphatidylethanolamine. Menaquinone-7 is the major respiratory quinone. The genome of the type strain has a size of 2.6 Mbp and a G+C content of 41.9 mol%. Genes putatively encoding for the complete light-harvesting bacteriorhodopsin system and biosynthesis of several further carotenoids occur in the genome.

The type strain is LEOWEIH-7C^T^ (=DSM 116390^T^ = JCM 36347^T^), isolated from lake Leopoldskroner Weiher (City of Salzburg, Austria).

The accession numbers of the 16S rRNA gene sequence and the genome sequence are OR064354 and JAVNWW000000000, respectively.

### Supplementary Information

Below is the link to the electronic supplementary material.

Supplementary file1 (PDF 420 KB)

## Data Availability

The Whole Genome Shotgun project has been deposited at DDBJ/ENA/GenBank under the accession JAVNWW000000000 for strain LEOWEIH-7C^T^ and JARDXH000000000 for strain LEPPI-3A. These are the versions described in this paper. The accession of the 16S rRNA gene sequence deposited at DDBJ/ENA/GenBank of strain LEOWEIH-7C^T^ is OR064354. All genomes in Table 2 are available in the IMG/MER system (ID numbers see Table 2).

## Abbreviations

A.: *Aquirufa*
gANI: Whole-genome average nucleotide identity
dDDH: Digital DNA–DNA hybridization
NSY medium: Nutrient broth soytone yeast extract medium
gyrB: B subunit of the DNA gyrase
GC/MS: Gas chromatography/mass spectrometry
IMG/MER: Integrated Microbial Genomes and Microbiomes Expert Review
AF: Alignment fraction
gAAI: Whole-genome average amino acid identity
*L.*: *Litoribacter*
