# Supplementary material for: Description of a new freshwater bacterium Aquirufa regiilacus sp. nov., classification of the genera Aquirufa, Arundinibacter, Sandaracinomonas, and Tellurirhabdus to the family Spirosomataceae, classification of the genus Chryseotalea to the family Fulvivirgaceae and Litoribacter to the family Cyclobacteriaceae, as well as classification of Litoribacter alkaliphilus as a later heterotypic synonym of Litoribacter ruber
Source: Arch Microbiol. 2024 Jan 27;206(2):79. doi: 10.1007/s00203-023-03801-8 (PMC10821818; doi:10.1007/s00203-023-03801-8)
Supplement: Supplementary file 1 — Supplementary file1 (PDF 420 KB) [file 203_2023_3801_MOESM1_ESM.pdf]

Supplementary Information

Archives of Microbiology

Description of a new freshwater bacterium *Aquirufa regiilacus* sp. nov., classification of the genera *Aquirufa*, *Arundinibacter*, *Sandaracinomonas* and *Tellurirhabdus* to the family *Spirosomataceae*, classification of the genus *Chryseotalea* to the family *Fulvivirgaceae* and *Litoribacter* to the family *Cyclobacteriaceae* as well as classification of *Litoribacter alkaliphilus* as a later heterotypic synonym of *Litoribacter ruber*

Alexandra Pitt, Stefan Lienbacher, Johanna Schmidt, Meina Neumann-Schaal, Jacqueline Wolf, and

Martin W. Hahn

Correspondence: alexandra.pitt@uibk.ac.at

Table S1. Fatty acid composition of strain LEOWEIH-7C^T. Only fatty acids with values ≥ 1 % were listed. Major fatty acids (> 10 %) were marked with bold letters.

Fatty acid	(%)
$C_{14:0}$	2.7
$C_{15:1}\omega 4c$ (Unknown 14.959)	4.4
$C_{15:1}\omega 6c$	3.2
$C_{16:1}\omega 5c$	9.1
**$C_{16:1}\omega 7c$**	**16.8**
$C_{17:1}\omega 6c$	1.3
iso-$C_{11:0}$	1.9
**iso-$C_{15:0}$**	**31.0**
**anteiso-$C_{15:0}$**	**11.0**
anteiso-$C_{15:1}\omega 7c$	1.7
Iso-$C_{15:1}\omega 5c$	1.8
Iso-$C_{15:1}\omega 7c$	1.8
Iso-$C_{17:1}\omega 5c$	1.6
Iso-$C_{17:1}\omega 7c$	1.2
Iso-$C_{15:0}$3-OH	3.7

Table S2. IMG/MER ID numbers of the mentioned genes from Table 2

1, LEOWEIH-7C[T]; 2, LEPPI-3A; 3, *A. antheringensis* 30S-ANTBAC[T]; 4, *A. lenticrescens* 9H-EGSE[T]

	1	2	3	4
IMG/MER ID number, genome	8023692520	8014893578	2816332120	2857132225
Genes predicted for:				
Bacteriorhodopsin (COG5524)	8023693089	8014894940	2816428628	2857132839
β-Carotene 15,15'-monooxygenase (TIGR03753)	8023693090	8014894939	2816428629	2857132840
Synthesis of β-carotene:	+	+	+	+
main gene: lycopene beta-cyclase (EC:5.5.1.19)	8023693091	8014894938	2816428630	2857132394
	8023693694	8014895711	2816430396	2857132841
Nitrate reductase, assimilatory (EC:1.7.7.2)	8023693629	8014895777	2816430450	-
Nitrite reductase, assimilatory (EC:1.7.1.15)	8023693633	8014895774	2816430446	-
	8023693632	8014895773	2816430447	
MFS transporter: nitrate/nitrite (COG2223)	8023693630	8014895776	2816430449	-
Catalase-peroxidase (EC:1.11.1.21)	8023694408	8014893703	2816429218	2857133430
Cytochrome c peroxidase (EC:1.11.1.5)	8023694358	8014893654	2816429064	2857133272
	8023692810	8014894600		
Cytochrome c oxidase:	+	+	+	+
main gene: subunit 1	8023694709	8014895822	2816428236	2857132455
Cytochrome c oxidase cbb3 type	8023693185	8014894844	2816429970	2857134213
	8023693187	8014894842	2816429972	2857134215
Endoglucanase, Cellulase (EC:3.2.1.4)	8023694447	8014893745	-	2857132826
Rubredoxin (COG1773)	8023693627	8014895779	2816430452	-
Genes involved in gliding motility	10	10	14	16
	8023692724	8014894010	2816430000	2857132960
	8023692719	8014895168	2816428736	2857132814
	8023693216	8014894813	2816429266	2857133323
	8023694807	8014893876	2816429499	2857133732
	8023693787	8014894690	2816429566	2857133812
	8023693825	8014894685	2816429560	2857133107
	8023694582	8014894689	2816429561	2857133814
	8023692720	8014895937	2816429562	2857132903
	8023692644	8014893838	2816430211	2857133292
	8023693959	8014894765	2816429078	2857133813
			2816428268	2857134244
			2816429095	2857133818
			2816428883	2857133308
			2816428678	2857133316
				2857133481
				2857132487
Genes for PorP/SprF proteins, type IX secretion system (TIGR03519)	6	6	5	6
	8023692643	8014893839	2816428600	2857132813
	8023692717	8014893877	2816429094	2857133307
	8023692918	8014893941	2816429498	2857133315

	8023693788	8014894492	2816429559	2857133322
	8023693826	8014894692	2816429743	2857133731
	8023693890	8014894766		2857133811

Figure S1 (next page). Reconstruction of the phylogenetic position of the investigated strains based on almost full-length 16S rRNA gene sequences (1319 alignment positions). Shown is a neighbour-joining midpoint rooted tree. At least all type species of the genera listed at LPSN (Meier-Kolthoff et al. 2021) of the families *Cytophagaceae*, *Spirosomataceae*, *Flexibacteriaceae* and *Fulvivirgaceae*, as well as a selection of the type species of the family *Cyclobacteriaceae* were included. The classification of the genera to families were taken from Figure 1. Bootstrap values above 50 % are indicated. *, no genome sequence available, bar, 0.02 substitutions per nucleotide position.

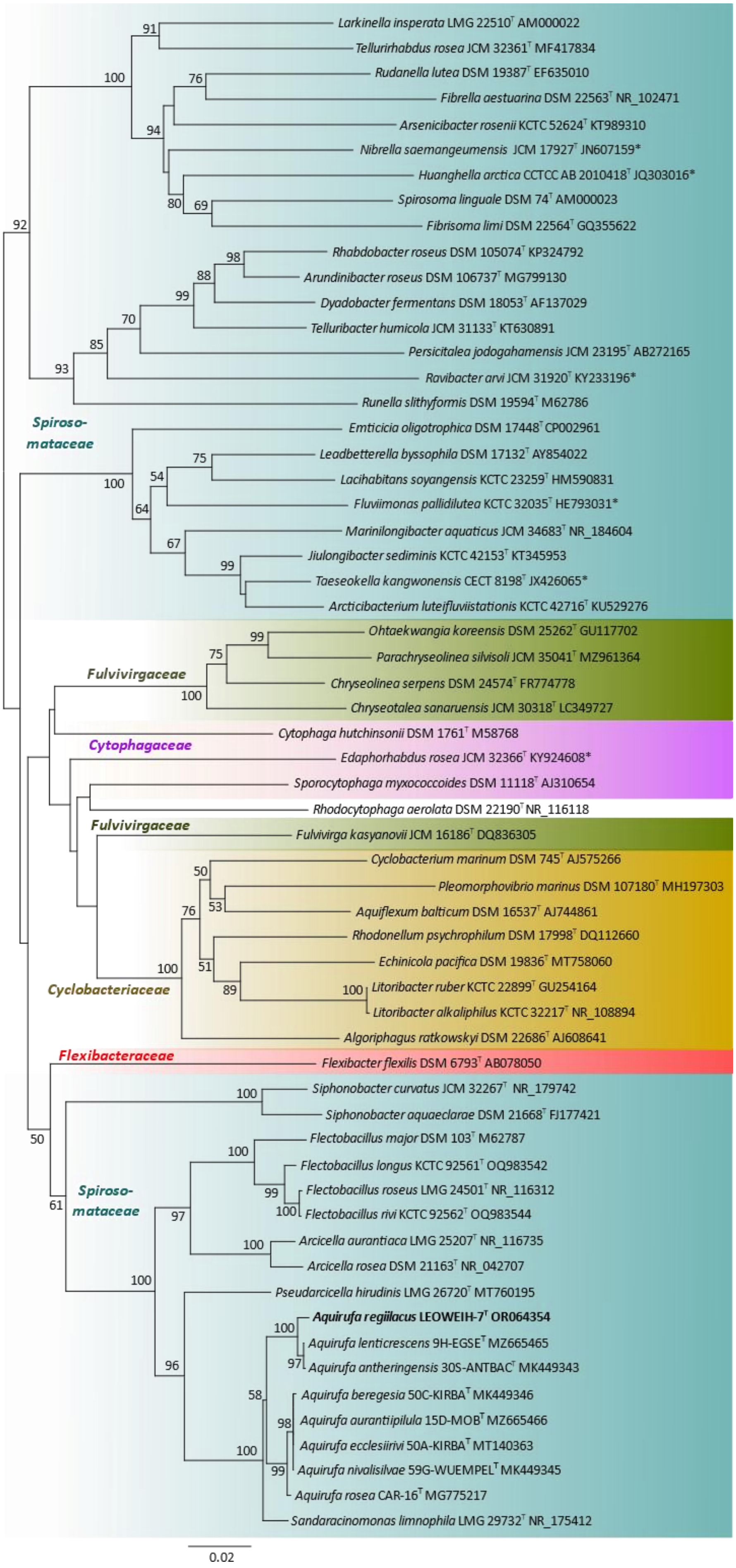
Larkinella insperata LMG 22510T AM000022
Tellurirhabdus rosea JCM 32361T MF417834
Rudanella lutea DSM 19387T EF635010
Fibrella aestuarina DSM 22563T NR_102471
Arsenicibacter rosenii KCTC 52624T KT989310
Nibrella saemangeumensis JCM 17927T JN607159*
Huanghella arctica CCTCC AB 2010418T JQ303016*
Spirosoma linguale DSM 74T AM000023
Fibrisoma limi DSM 22564T GQ355622
Rhabdobacter roseus DSM 105074T KP324792
Arundinibacter roseus DSM 106737T MG799130
Dyadobacter fermentans DSM 18053T AF137029
Telluribacter humicola JCM 31133T KT630891
Persicitalea jodogahamensis JCM 23195T AB272165
Ravibacter arvi JCM 31920T KY233196*
Runella slithyformis DSM 19594T M62786
Spiroso-mataceae
Emticicia oligotrophica DSM 17448T CP002961
Leadbetterella byssophila DSM 17132T AY854022
Lacihabitans soyangensis KCTC 23259T HM590831
Fluviimonas pallidilutea KCTC 32035T HE793031*
Marinilongibacter aquaticus JCM 34683T NR_184604
Jiulongibacter sediminis KCTC 42153T KT345953
Taeseokella kangwonensis CECT 8198T JX426065*
Arcticibacterium luteifluviistationis KCTC 42716T KU529276
Ohtaekwangia koreensis DSM 25262T GU117702
Parachryseolinea silvisoli JCM 35041T MZ961364
Fulvivirgaceae
Chryseolinea serpens DSM 24574T FR774778
Chryseotalea sanaruensis JCM 30318T LC349727
Cytophaga hutchinsonii DSM 1761T M58768
Cytophagaceae
Edaphorhabdus rosea JCM 32366T KY924608*
Sporocytophaga myxococcoides DSM 11118T AJ310654
Rhodocytophaga aerolata DSM 22190T NR_116118
Fulvivirgaceae
Fulvivirga kasyanovii JCM 16186T DQ836305
Cyclobacterium marinum DSM 745T AJ575266
Pleomorphovibrio marinus DSM 107180T MH197303
Aquiflexum balticum DSM 16537T AJ744861
Rhodonellum psychrophilum DSM 17998T DQ112660
Echinicola pacifica DSM 19836T MT758060
Litoribacter ruber KCTC 22899T GU254164
Cyclobacteriaceae
Litoribacter alkaliphilus KCTC 32217T NR_108894
Algoriphagus ratkowskyi DSM 22686T AJ608641
Flexibacteraceae
Flexibacter flexilis DSM 6793T AB078050
Siphonobacter curvatus JCM 32267T NR_179742
Siphonobacter aquaeclarae DSM 21668T FJ177421
Flectobacillus major DSM 103T M62787
Flectobacillus longus KCTC 92561T OQ983542
Flectobacillus roseus LMG 24501T NR_116312
Spiroso-mataceae
Flectobacillus rivi KCTC 92562T OQ983544
Arcicella aurantiaca LMG 25207T NR_116735
Arcicella rosea DSM 21163T NR_042707
Pseudarcicella hirudinis LMG 26720T MT760195
Aquirufa regiilacus LEOWEIH-7T OR064354
Aquirufa lenticrescens 9H-EGSET MZ665465
Aquirufa antheringensis 30S-ANTBACT MK449343
Aquirufa beregesia 50C-KIRBAT MK449346
Aquirufa aurantiipilula 15D-MOBT MZ665466
Aquirufa ecclesiirivi 50A-KIRBAT MT140363
Aquirufa nivalisilvae 59G-WUEMPELT MK449345
Aquirufa rosea CAR-16T MG775217
Sandaracinomonas limnophila LMG 29732T NR_175412
0.02

Figure S2. Polar lipid pattern of strain LEOWEIH-7C^{T}.

First row: left side, visualisation of total lipids with dodecamolybdophosphoric acid; right side: visualisation of glycolipids with α-naphthol

Second row: left side, visualisation of phospholipids with molybdenum blue; right side, visualisation of aminolipids with ninhydrin.

PE, phosphatidylethanolamine; APL, aminophospholipid; AL, aminolipid; L, lipid

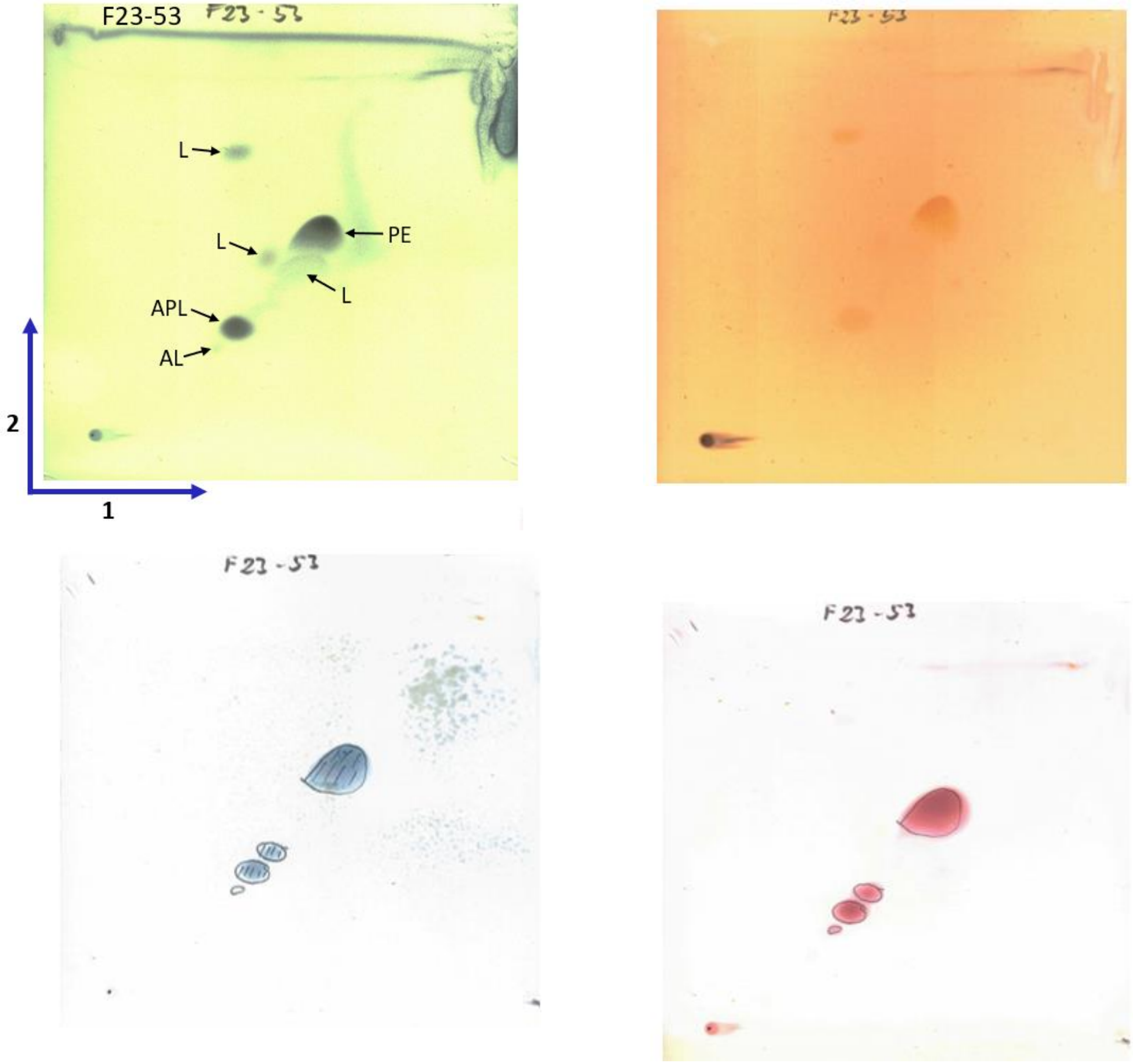

References:

Meier-Kolthoff JP, Carbasse JS, Peinado-Olarte RL, Göker M (2021) TYGS and LPSN: a database tandem for fast and reliable genome-based classification and nomenclature of prokaryotes. Nucleic Acids Res 50:D801-D807. https://doi.org/10.1093/nar/gkab902
